# A collaborative approach to adopting/adapting guidelines. The Australian 24-hour movement guidelines for children (5-12 years) and young people (13-17 years): An integration of physical activity, sedentary behaviour, and sleep

**DOI:** 10.1186/s12966-021-01236-2

**Published:** 2022-01-06

**Authors:** Anthony D. Okely, Davina Ghersi, Sarah P. Loughran, Dylan P. Cliff, Trevor Shilton, Rachel A. Jones, Rebecca M. Stanley, Julie Sherring, Natalie Toms, Simon Eckermann, Timothy S. Olds, Zhiguang Zhang, Anne-Maree Parrish, Lisa Kervin, Sandra Downie, Jo Salmon, Clair Bannerman, Tamie Needham, Elaine Marshall, Jordy Kaufman, Layne Brown, Janecke Wille, Greg Wood, David R. Lubans, Stuart J. H. Biddle, Shane Pill, Anthea Hargreaves, Natalie Jonas, Natasha Schranz, Perry Campbell, Karen Ingram, Hayley Dean, Adam Verrender, Yvonne Ellis, Kar Hau Chong, Dorothea Dumuid, Peter T. Katzmarzyk, Catherine E. Draper, Hayley Lewthwaite, Mark S. Tremblay

**Affiliations:** 1 Faculty of Arts, Social Sciences and Humanities, School of Health and Society, University of Wollongong, Wollongong, NSW 2522 Australia; 2 Illawarra Health and Medical Research Institute, Wollongong, Australia; 3 Research Policy and Translation, National Health and Medical Research Council, Canberra, Australia; 4 National Health & Medical Research Council Clinical Trials Centre, Sydney Medical School, University of Sydney, Sydney, Australia; 5 National Heart Foundation (WA), 334 Rokeby Road, Subiaco, Australia; 6 Preventive Programs, Commonwealth Department of Health, Canberra, Australia; 7 Australian Health Services Research Institute, University of Wollongong, Wollongong, Australia; 8 Alliance for Research in Exercise, Nutrition and Activity (ARENA), Allied Health and Human Performance, University of South Australia, Adelaide, Australia; 9 Institute for Physical Activity and Nutrition (IPAN), Deakin University, Melbourne, Australia; 10 Department of Education, Canberra, Australia; 11 Department of Health, Darwin, NT Australia; 12 Department of Health, Tasmania, Australia; 13 Swinburne University of Technology, Melbourne, Australia; 14 Federation of Ethnic Communities Council of Australia (FECCA), Canberra, Australia; 15 Australian Sports Commission, Leederville, Western Australia; 16 Priority Research Centre for Physical Activity and Nutrition, School of Education, University of Newcastle, Newcastle, Australia; 17 Centre for Health Research, University of Southern Queensland, Springfield Central, Toowoomba, Australia; 18 The Australian Council for Health, Physical Education and Recreation (ACHPER), Wayville, Australia and Flinders University, Adelaide, South Australia; 19 Cycling and Walking Australia New Zealand, Whanganui, New Zealand; 20 Australian Curriculum, Assessment and Reporting Authority (ACARA), SA, Sydney, Australia; 21 Active Healthy Kids Australia, Adelaide, Australia and National Heart Foundation, Adelaide, South Australia; 22 Australian Children’s Education & Care Quality Authority (ACECQA), Sydney, Australia; 23 NSW Education Standards Authority (NESA), Sydney, Australia; 24 Pennington Biomedical Research Center, Louisiana, USA; 25 SAMRC/Wits Developmental Pathways for Health, University of the Witwatersrand, Johannesburg, South Africa; 26 Healthy Active Living and Obesity Research Group, Children’s Hospital of Eastern Ontario Research Institute, Ottawa, Canada

**Keywords:** Methodology, GRADE-ADOLOPMENT, Public health recommendations, Guideline development

## Abstract

**Abstract:**

**Background:**

In 2018, the Australian Government updated the Australian Physical Activity and Sedentary Behaviour Guidelines for Children and Young People. A requirement of this update was the incorporation of a 24-hour approach to movement, recognising the importance of adequate sleep. The purpose of this paper was to describe how the updated *Australian 24-Hour Movement Guidelines for Children and Young People (5 to 17 years): an integration of physical activity, sedentary behaviour and sleep* were developed and the outcomes from this process*.*

**Methods:**

The GRADE-ADOLOPMENT approach was used to develop the guidelines. A Leadership Group was formed, who identified existing credible guidelines. The *Canadian 24-Hour Movement Guidelines for Children and Youth* best met the criteria established by the Leadership Group. These guidelines were evaluated based on the evidence in the GRADE tables, summaries of findings tables and recommendations from the Canadian Guidelines. We conducted updates to each of the Canadian systematic reviews. A Guideline Development Group reviewed, separately and in combination, the evidence for each behaviour. A choice was then made to adopt or adapt the Canadian recommendations for each behaviour or create *de novo* recommendations. We then conducted an online survey (n=237) along with three focus groups (n=11 in total) and 13 key informant interviews. Stakeholders used these to provide feedback on the draft guidelines.

**Results:**

Based on the evidence from the Canadian systematic reviews and the updated systematic reviews in Australia, the Guideline Development Group agreed to adopt the Canadian recommendations and, apart from some minor changes to the wording of good practice statements, maintain the wording of the guidelines, preamble, and title of the Canadian Guidelines. The Australian Guidelines provide evidence-informed recommendations for a healthy day (24-hours), integrating physical activity, sedentary behaviour (including limits to screen time), and sleep for children (5-12 years) and young people (13-17 years).

**Conclusions:**

To our knowledge, this is only the second time the GRADE-ADOLOPMENT approach has been used to develop movement behaviour guidelines. The judgments of the Australian Guideline Development Group did not differ sufficiently to change the directions and strength of the recommendations and as such, the Canadian Guidelines were adopted with only very minor alterations. This allowed the Australian Guidelines to be developed in a shorter time frame and at a lower cost. We recommend the GRADE-ADOLOPMENT approach, especially if a credible set of guidelines that was developed using the GRADE approach is available with all supporting materials. Other countries may consider this approach when developing and/or revising national movement guidelines.

**Supplementary Information:**

The online version contains supplementary material available at 10.1186/s12966-021-01236-2.

## Background

The first National Physical Activity Recommendations for Children and Adolescents were released by the Australian Government in 2004 [1]. These were updated in 2012 and, for the first time, included separate sedentary behaviour guidelines for the same age group [2, 3]. In recent years, guidelines have evolved to accommodate – from a movement perspective – the entire day [4]. This perspective is called 24-hour integrated movement guidelines [5], and acknowledge that individual movement behaviours – physical activity, sedentary behaviour and sleep – need to be considered in combination with one other when examining their associations with health in children and young people. In 2016, Canada released the first integrated 24-hour movement guidelines for school-age children and youth [5]. The evidence underpinning these guidelines showed a monotonic relationship between the number of movement behaviour guidelines met by an individual and associated health indicators [6–8]. That is, meeting all three guidelines was better than meeting any two, and meeting any combination of two guidelines was better than meeting just one, which in turn was better than meeting none. In early 2018, the Australian Government provided funding to update the Australian Physical Activity and Sedentary Behaviour Guidelines for Children and Young People, with the request that these be 24-hour movement guidelines. The benefit for Australia was leveraging the significant work completed in Canada on the development of their 24-hour guidelines resulting in the process requiring considerably less time and fewer resources. The benefits of adapting guidelines produced by others was something Australia had successfully done with their 24-hour movement behaviour guidelines for the early years [9].

The GRADE-ADOLOPMENT approach allows guideline developers to follow the GRADE process for developing guidelines more eficiently by adapting or adopting an existing evidence-based guidelines [10]. This approach prevents the need to undertake (or repeat) resource and time-intensive tasks such as conducting full systematic reviews. It also allows local guideline developers to take local contextual factors into consideration.

Based on the Canadian Guideline Development Panel’s use of the GRADE approach to develop the *Canadian 24-Hour Movement Guidelines for Children and Youth*, the GRADE-ADOLOPMENT approach was used in the development of the *Australian 24-Hour Movement Guidelines for Children and Young People*. The purpose of this paper was to describe how GRADE-ADOLOPMENT approach was used to develop the *Australian 24-Hour Movement Guidelines for Children and Young People*. This process started in May 2018 and was completed in December 2018, with the Guidelines released in April 2019.

## Methods

### Guideline ADOLOPMENT structure

**Fig. 1:**
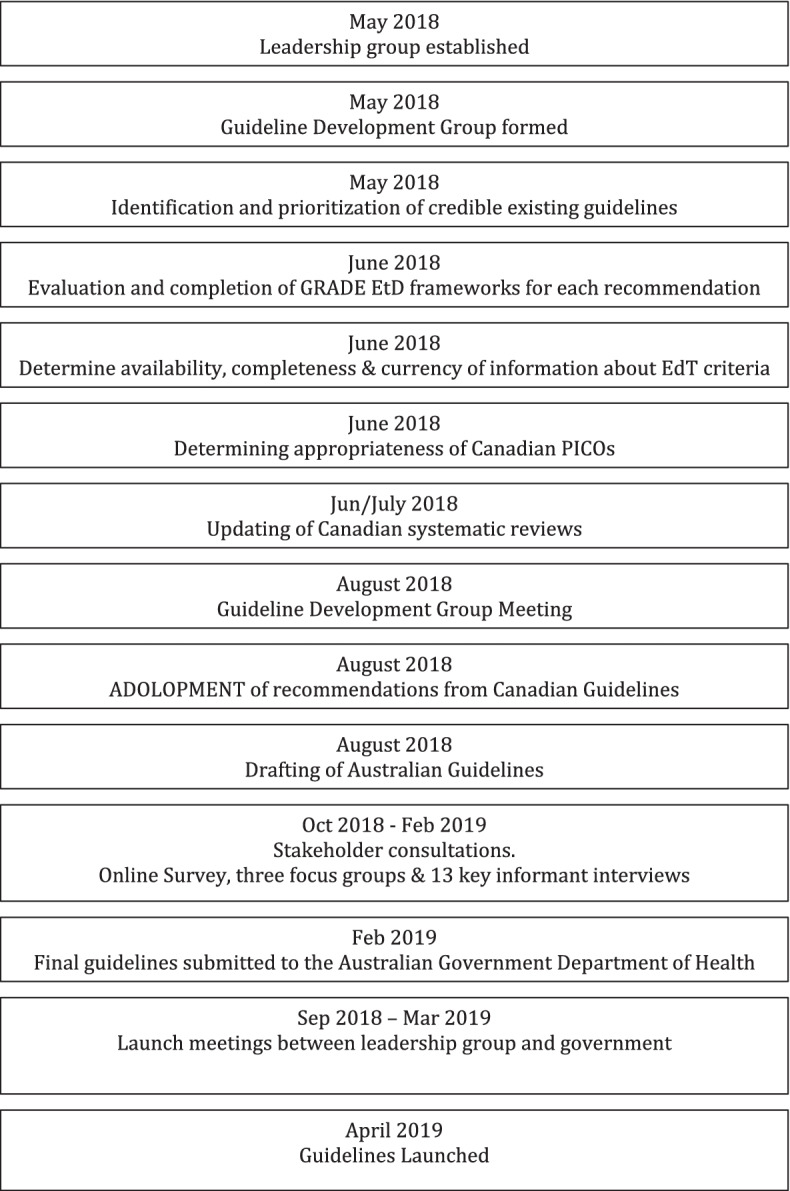
Timeline and sequence of events involved in the development of the Australian 24-hr movement guidelines for children and young people: an integration of physical activity, sedentary behaviour, and sleep

The GRADE-ADOLOPMENT process followed the framework described in detail by Schünemann and colleagues [10]. Several steps that were identified in the Appraisal of Guidelines for Research & Evaluation II (AGREE-II) instrument [11] were added by the Leadership Group. A summary of the timeline and sequence of steps used is shown in Fig. 1.

- *Step 1: Establishment of a Leadership Group.* This group comprised the project Principal Investigators (ADO, SPL, DPC, AMP, TSO, LK, SE, RAJ, RMS, MST), a guideline methodologist (DG), and representatives from the Australian Government (owner and funder of the Guidelines; SD, NT), National Heart Foundation of Australia (key stakeholder; TS), and professional support from Early Start at the University of Wollongong (JS/YGE). This group was formed in April 2018 and met fortnightly up to the end of August 2018 to provide strategic advice and direction, guidance, and budget accountability to the project. Ad-hoc subcommittees were formed for the areas of stakeholder consultation (RAJ, RMS, JS), communication and dissemination (TS, SD, NT, JS, ADO) and surveillance (ADO, JS, NS, EM, SD, NT) at appropriate time points in the process. As the Australian guidelines sought to adopt or adapt the Canadian Guidelines using the GRADE-ADOLOPMENT process (assuming these would be appropriate as per Step 3 – see below for details), it was agreed that the Principal Investigator from the Canadian Guidelines (MST) would be part of the leadership group.
- *Step 2: Formation of a Guideline Development Group.* A Guideline Development Group (GDG) was formed which included additional expert researchers, representatives from key stakeholder groups (including parents and Indigenous Australian communities), and methodology experts (Table S1). The role of the GDG is described in detail in Step 5. Efforts were made to achieve geographical representation across Australia within the confines of the budget.
- *Step 3: Identification of credible existing guidelines and definition of criteria for selection of the guidelines.* We were aware of two sets of 24-hour integrated movement guidelines for children and young people. These were from Canada [12] and New Zealand [13]. The New Zealand Guidelines adopted those from Canada. The Canadian 24-hour Movement Guidelines were considered along with other existing integrated or physical activity and sedentary behaviour guidelines that met the following criteria: 1) published in the past five years (or in the process of being published); 2) addressed clear research questions (contained all Population, Intervention, Comparator and Outcome [PICO] elements); 3) followed the GRADE process; 4) allowed for updating (provided access to full systematic reviews, which were registered with the Prospective Register of Systematic Reviews (PROSPERO) and provided full access to the search strategy); 5) included existing and accessible GRADE tables and summaries of findings; and 6) completed a risk-of bias assessment [10]. Table 1 contains a summary of the national physical activity and sedentary behaviour guidelines in children and young people that the leadership group was able to identify and the evaluation of each against these criteria. Only the 2016 Canadian 24-Hour Movement Guidelines for Children and Youth met all criteria and were therefore chosen as the guidelines to be adopted or adapted following the GRADE-ADOLOPMENT process.

**Table 1:** Existing international Physical Activity Guidelines for Children and Young People

Criteria	USA 2018^a^	China 2017	Chile 2017	Netherlands 2017	New Zealand 2017	Canada 2016	France 2016	Germany 2016	Norway 2016	Mexico 2015	Spain 2015	Australia 2014	Austria 2013	Denmark 2014	Paraguay 2014	Turkey 2014	Qatar 2014	Argentina 2013
Followed GRADE process	N	N	?	N	Y	Y	N	N	?	Y	Y	Y	N	N	Y	N	N	Y
Addresses clear questions (can identify PICO elements)	Y	N	?	N	Y	Y	Y	?	?	Y	Y	Y	N	?	Y	N	N	Y
Has benefits and harms assessments	Y	N	?	Y	Y	Y	Y	Y	Y	Y	Y	Y	Y	?	Y	Y	Y	Y
Assessed using AGREE	N	N	?	N	Y	Y	N	N	?	N	N	Y	N	N	N	N	N	N
Allows for updating	?	N	?	Y	N	Y	Y	N	?	Y	Y		N	?	Y	N	N	Y
Has existing and accessible evidence tables /summaries	Y	N	?	Y	Y	Y	Y	Y	?	N	Y	Y	Y	?	Y	N	N	Y
Has risk of bias assessment	Y	N	?	Y	Y	Y	N	?	?	Y	Y	Y	N	?	Y	N	N	Y
Were integrated (24hr)	?	N	?	N	Y	Y	N	N	?	N	N	N	N	?	N	N	N	N
Costs associated with implementing guideline	N	?	?	?	N	Y	N	N	?	?	?	N	N	?	?	?	?	?
Acccompanying – how they are going to implement – disseminate the guidelines	N	N	?	?	N	Y	Y	N	?	?	?	N	Y	?	?	?	?	?

Reference: Appendix 1. GRADE-ADOLOPMENT (Schünemann et al., J Clin Epidemiol. 2017)

^a^under development during guideline development process but made available to Australian Guideline Development Group

Key: Y=yes; N=no; ?=unsure

The AGREE-II tool was used to determine the credibility of the Canadian Guidelines (as per Stage 1 of the suggested GRADE-ADOLOPMENT Protocol – see Appendix 1 [10]. Following the credibility assessment, the ADOLOPMENT framework moves on to the evaluation and final selection of the guidelines that will be adopted or adapted. It was agreed by the Leadership Group that it would be appropriate to adopt the Canadian Guidelines as they were determined to be of appropriate quality, their scope/applicability was appropriate for Australia, the topic was a priority for Australia and the research questions and PICOs (Population, Intervention, Comparators, and Outcomes) for the systematic reviews that served as the evidence base were relevant.

- *Step 4: Evaluate and complete GRADE Evidence-to-Decision (EtD) frameworks for each recommendation.* The Australian Guideline Development Group considered the evidence-to-decision criteria that influenced the direction and strength of each of the draft recommendations made by the Canadian Guideline Development Panel. These were based on the GRADE tables, summary of findings tables, and recommendations made available by the Canadian Guideline Leadership Committee.

Assessed against the stated GRADE approach to evidence synthesis (i.e., 60% of randomised controlled trials [RCTs] were statistically significant and positive), the evidence base was graded “Low” or “Very Low” in most cases. The Guideline Development Group then made a decision to support or not support the 2016 Canadian Guidelines based on the evidence and other criteria used to make recommendations including values and preferences; feasibility, acceptability and equity issues; resources; balance of benefits and harms; and quality of the evidence [11]. Parts of the EtD framework that were able to be followed during the Guideline Development Group meeting included presenting the evidence and keeping track of the discussion and judgments. Following the Guideline Development Group meeting, a transparent record of the discussions was communicated to those who attended for verification.

- *Step 5 Determine availability, completeness, and currency of information about EtD criteria.* The next component in the general stages of GRADE-ADOLOPMENT (see Appendix 2 [10]) was to determine the availability, completeness, and currency of the information about the EtD criteria. For this, the criteria for updating reviews found in Appendix 4 of the GRADE-ADOLOPMENT paper [10] was used (see Table 2). Based on this information, the Leadership Group made a decision to update the Canadian systematic reviews focusing only on the critical outcomes (see [14] for a list of these for each systematic review) for randomized controlled trials and cohort study designs because the sources of these reviews were older than three months (i.e., they had an end date before February 2018) [10]. The exception was the systematic review for the combinations of movement behaviours. Because there were fewer studies in this area it was decided to also include cross-sectional studies in this systematic review. The Leadership Group decided not to update the reviews for non-critical outcomes (see [14] for a list of these) or for cross-sectional studies because the consensus was that even if an update was to uncover new studies, they would be graded very-low to low quality and as such, would not result in a change to the final guidelines, and there were already many such studies that were used to inform the guidelines.

**Table 2:** Criteria for updating reviews

Criterion	Minor update (all criteria must apply)
Prior Review (for question)	A credible systematic review exists
Full text reviewed for the Research Question of interest	≤20 articles
New Studies	≤5 studies
Evidence profile available?	Available
Outcomes all addressed	All important outcomes addressed

Reference: Appendix 4: GRADE-ADOLOPMENT [10].

The Australian Leadership Group made the PICOs that guided the four systematic reviews for the 2016 Canadian Guidelines available for comment by the Australian Guideline Development Group prior to the Consensus meeting. This latter group was asked to comment on the appropriateness of each of the PICOs for the Australian context. Some of the initial comments sought clarification on the selection of the specific search terms for some of the outcomes. These comments were resolved by indicating that the search terms would be or were captured in the Australian or Canadian searches, respectively, although this information was not clear in the PICOs. Other queries related to the inclusion of information in the summary tables or in the PROSPERO registration or to the definitions of specific terms. Where changes were suggested, these were discussed by the Leadership Group and agreement reached. None of the proposed changes were substantial enough to warrant changing any of the existing PICOs except for the sedentary behaviour PICO. The Australian Leadership group decided to include “psychological distress” (which included stress, anxiety symptoms, depressive symptoms, and mental health) as an additional critical outcome and to move the outcome of “self-esteem” from an important to critical outcome. In addition, two additional considerations were made to all the systematic reviews. These were to: 1) consider and discuss *cost-effectiveness* and *resource use* as per the GRADE-ADOLOPMENT approach and in the context of the proposed Guideline recommendation; and 2) use the evidence to seek to address the applicability of the recommendations to Indigenous Australians and their communities.

The updates to the four systematic reviews initially performed for the Canadian Guidelines were conducted with searches completed up to the end of July 2018. For each systematic review, the quality of evidence was assessed by outcome/indicator, study design, and age group, using the GRADE approach [15, 16]. Each systematic review used the same PICO as the corresponding systematic review completed for the 2016 Canadian Guidelines [17–20].

The results of these systematic review updates were presented at the Guideline Development Group meeting from 22-23 August 2018. The specific objectives of this meeting were to review, discuss, debate and interpret findings from the Canadian systematic reviews and Australian updated searches, including compositional analyses that were performed using data from Canada and Australia. Other objectives were to review and adopt/adapt the *Preamble* and the actual *Canadian 24-Hour Movement Guidelines for Children and Youth*; discuss proposed stakeholder consultations; identify research gaps; and plan the launch, dissemination, promotion, integration, and evaluation activities for the *Australian 24-hour Movement Guidelines for Children and Young People.*

The process at the Guideline Development Group meeting involved reviewing the evidence for each movement behaviour (physical activity, sedentary behaviour, and sleep) individually, starting with the 2016 Canadian systematic reviews and integrating the Australian updates into these reviews. The evidence for each behaviour, including the conclusions of the Canadian review and how this process informed their guidelines, was then discussed. The Guideline Development Group then followed the GRADE-ADOLOPMENT process to decide to adopt or adapt the 2016 Canadian recommendations for each behaviour or create *de novo* recommendations. In addition, the Panel examined the results of the integrated behaviours systematic review and compositional data analyses from Canada [21], infused expert opinion into the evidence (such as feasibility, acceptability, equity issues, values and preferences, resources, and balance of benefits and harms), and combined evidence of absolute effects across multiple outcomes [22–25]. This led to an informed assessment of whether the panel either agreed or disagreed with the judgements made by the Canadian Guideline Development Panel. If the Australian Guideline Development Group agreed with the judgements, the recommendations were adopted, and the Panel moved on to discuss the wording of the guidelines. If the Panel disagreed with the judgements, the recommendations were adapted, and the Panel moved on to describe the reasons for deviation in the EtD framework. It was noted during the Guideline Development Group meeting that a recommendation could be adopted and still added to or translated for adoption in the wording and adjusted if necessary, based on this detailed discussion.

The next three sections of the Guideline Development Process [26] are not components of the GRADE-ADOLOPMENT process but were important when assessing the appropriateness of the adopted guidelines with key stakeholders and the development of plans for the Australian Government (owner of the Guidelines) to consider for promotion and activation of the Guidelines and potential monitoring and surveillance. This process was also followed in updating the Australian 24-hr Movement Guidelines for the Early Years [9].

### Stakeholder consultations

The online survey developed as part of the 2016 Canadian Guidelines [14] was modified for the Australian context to seek feedback from stakeholders regarding their level of agreement with the draft Australian Guidelines which eminated from the Guideline Development Group meeting. The Human Research Ethics Committee of the University of Wollongong approved the administration of the survey and use of a passive consent process (HE 2018/370). The survey sought feedback regarding the clarity of the title, preamble, and guidelines as well as levels of agreement with the text. Basic demographic information was requested, and respondents were afforded the opportunity to provide comments on all components of the guidelines. Guideline Development Group members were asked to disseminate the survey through their networks and used a snowball sampling methodology to optimise reach and input from relevant stakeholders. The survey was open from September 17 to October 29, 2018. After the survey closed, numerical responses from participants were tabulated and analysed. Written comments were consolidated into themes and summaries were prepared. The stakeholder survey also allowed respondents to express their interest in publicly disclosing their support for the guidelines pending their review of the final draft. To facilitate this, interested respondents were asked to provide an email address where the final guidelines could be sent.

In addition, focus groups (conducted in person) and key informant interviews (in person and remotely) were conducted. These targeted key stakeholders who were difficult to reach through the online survey, such as parents of varying socioeconomic status and cultural backgrounds – in particular Australian Indigenous and low-socioeconomic groups. Specific questions about the acceptability and perceived importance, clarity of the Guidelines and preamble, facilitators and barriers to implementation and dissemination, and dissemination and implementation recommendations for the Guidelines were asked. The focus groups were supplemented with key informant interviews held with a culturally and linguistically diverse parent; sports coach; teacher and policy maker from the disability sector; principal of a school located in a low socioeconomic area; school counsellor; policy maker from the education sector; after-school director and teacher; Australian Indigenous young person; Active Healthy Kids Australia Project Officer; and an Australian Indigenous parent. A total of 11 individuals participated in three focus groups and thirteen interviews (1 participant per interview). Recruitment occurred through existing partnerships and connections. Focus groups and interviews lasted between 30 and 90 mins and were conducted from October 2018 to February 2019 in New South Wales, Australian Capital Territory, Tasmania, South Australia, Victoria and Western Australia by a member of the guideline development group from their state/territory. The focus groups and interviews were audio-recorded and transcribed verbatim and inductive thematic data analyses by two researchers were employed and consensus reached on any discrepancies through discussion [27]. Ethics approval was obtained from Human Research Ethics Committee of the University of Wollongong (HE 2018/370). A subcommittee of the Guideline Development Group reviewed the survey, focus group and key informant interview results, and suggested revisions to the Guidelines based on the stakeholder feedback, ensuring changes remained true to the available evidence base. Revisions agreed upon by the Leadership Group were then circulated to the entire Guideline Development Group for comment and final revisions. Consensus was achieved on the final Guidelines.

## Results

### Updates to systematic reviews

The results of the updates to the Canadian systematic reviews by the Australian Leadership Group are described below.

#### Physical activity

For physical activity, 5,085 new studies were identified from a search of databases, with 132 studies remaining after screening title and abstracts. Of these, 42 studies met the criteria to be included in the update.

Eleven studies examined the relationship between physical activity and body composition.

Two studies used an RCT design, four studies used a non-randomized trial (NRT) design, and the remaining five studies used a longitudinal design. Among the two RCT’s, one study reported a mix of favourable and null findings [28], whereas the other study showed no intervention effect on body composition [29]. From the four NRT studies, one reported null effects of a physical activity intervention on adiposity outcomes [30]. The remaining three studies reported significant favourable effects on adiposity outcomes [31–33]. Among the five longitudinal studies, favourable associations between physical activity and body composition were reported [34–37]. One longitudinal study reported a mix of favourable and null associations for total physical activity and body composition [38].

Nine studies examined the relationship between physical activity and cardiometabolic biomarkers. One study used an NRT design; this study found significant favourable intervention effects on systolic blood pressure, total cholesterol and fasting glucose [30]. Among the eight longitudinal studies, six showed a favourable relationship between total physical activity, moderate- to vigorous-intensity physical activity (MVPA), moderate-intensity physical activity (MPA) and cardiometabolic biomarkers [35, 37, 39–42]. Two studies showed no relationship between total physical activity and cardiometabolic biomarkers [43, 44].

Six studies examined the relationship between physical activity and fitness. One study used an RCT design. This study reported a favourable effect on aerobic fitness at post-test [45]. Five studies used a NRT design. Of these, three studies showed a favourable effect on components of health-related fitness among those in the intervention group compared with the control group [33, 46–48]. One study reported mixed effects from a physical activity intervention on aerobic fitness across subsamples at post-test (favourable effect for Grade 6 children but not Grades 1 to 5) [49]. One study reported a favourable effect on endurance, co-ordination and shoulder mobility [32]. One longitudinal study showed a favourable, dose-response gradient between vigorous-intensity physical activity (VPA) and aerobic fitness [50].

Three studies examined the relationship between physical activity and behavioural conduct/pro-social behaviour. One RCT showed no effect from an intervention to increase MVPA on time in play and social skills [51]. One NRT showed there were positive effects of MVPA on effort and time on task [52]. One longitudinal study reported that physical activity was associated with fewer peer problems, but also that MVPA was unfavourably associated with hyperactivity problems (boys and girls) and conduct problems (boys only) [53].

Eleven studies examined the relationship between physical activity and cognition/academic achievement. Four were RCTs; three of these found positive effects on on-task behaviour [54–56]. Two studies found no intervention effect on content recall [57] or standardized test performance [56]. One study found no change on mathematical test performance following a physical activity intervention [56]. Four studies used a NRT design. Two of these showed a positive effect on on-task behaviour [58, 59] and one showed no effect [60]. Two studies showed no effect on sustained attention or executive functions (processing speed, selective attention) [60, 61]. Among the six longitudinal studies, four showed no relationships between physical activity and academic achievement [62–64] cognition [65, 66], or mathematics engagement [64, 67]. Four studies showed mixed relationships between physical activity and academic achievement [64], cognition [66] and mathematics engagement [67]. One study found unfavourable associations between light-intensity physical activity (LPA) and cognition [68].

Two studies examined the relationship between physical activity and harm/injuries. Both studies used a longitudinal design [69, 70]. The results were mixed, with one study showing that total, LPA, and VPA were negatively related to spinal pain [70], whereas the other study showed no relationship with spinal pain [69].

Overall, most of the updated studies showed that total physical activity was favourably associated with different health indicators (adiposity, cardiometabolic biomarkers, fitness, cognitive development and behavioural conduct/pro-social behaviour). The assessed quality of overall evidence using GRADE criteria for these outcomes did not change by including these additional studies from the updated review.

#### Sedentary behaviour

The sedentary behaviour updated systematic review captured 15,953 new studies with 286 studies remaining after titles and abstracts were screened. Of these, 34 studies met the criteria to be included in the update.

Fifteen studies examined the associations between sedentary behaviour and body composition. One study used a group NRT design, and 14 studies used a longitudinal design. The group NRT (n=41) showed no effect on total sitting time (during school time or over the whole day), although sitting in long bouts (>10 min) decreased and the number of sit-to-stand transitions increased as a result of the intervention [71]. However, the effects on body mass index and waist circumference z-scores were not statistically significant. The 14 longitudinal studies included 22,565 participants aged between 7 and 15 years. Eight of these studies found that higher durations or frequencies of accelerometer-derived sedentary time [72, 73] screen time [74–77], TV viewing [78] and weekend internet use [79] were significantly associated with less favourable body composition. One study reported that increased weekend TV was associated with moving between healthy weight and overweight categories between waves 1-3 (ages 4-5 years to 6-7 years). However, associations for computer use (weekday or weekend) or weekday TV were not associated with changes in weight category at any wave (2, 3 or 4), nor were changes in weekend TV between waves 1-2 or 1-4 [80]. Four studies reported no associations with indices of body composition [38, 42, 81, 82]. One study found that higher levels of device-measured sedentary behaviour were associated with better body composition in 454, 10-yr old children [35].

Six longitudinal studies examined the associations between sedentary behaviour and metabolic syndrome/cardiovascular disease risk factors. Three of these studies reported a dose-response gradient; higher screen time and higher sedentary time were associated with higher cardiometabolic risk [40, 41, 75]. The remaining studies showed a negative or null association between screen time, sedentary time and blood pressure/cardiometabolic risk factors [35, 42, 43].

Four studies examined the relationship between sedentary behaviour and behavioural conduct/pro-social behaviour. All were longitudinal in design and found that higher levels of non-specified screen time [83, 84], TV viewing [85] and video game use [86] were associated with unfavourable behavioural conduct/pro-social behaviour.

Six longitudinal studies examined the relationship between sedentary behaviour and academic achievement. Four of these found that higher levels of total screen time [86–88], and higher levels of non-school sedentary time excluding TV [89], were associated with lower academic achievement. Conversely, higher levels of device-measured sitting time, reading and homework outside of school were associated with higher academic achievement [66, 68], and more time spent in homework outside of school [68].

One longitudinal study examined the relationship between sedentary behaviour and self-esteem [90]. This study reported that in boys, higher levels of screen time were associated with lower self-esteem. Conversely, in girls, higher levels of TV viewing were associated with higher self-esteem.

Six longitudinal studies reported on the relationship between sedentary behaviour and psychological distress [91–96]. Four of these studies showed that higher levels of screen time were associated with higher levels of psychological distress [91, 92, 94, 95].

The assessed GRADE quality of overall evidence did not change for longitudinal studies examining adiposity (“Very Low”) or for RCTs examining psychosocial health (“Moderate”).

#### Sleep

For the updated sleep systematic review, 2,764 new studies were identified from the search of databases, with 1956 studies remaining after screening title and abstracts. A total of 21 additional studies met the inclusion criteria for the update.

One longitudinal study reported a significant unfavourable association between short sleep duration and adiposity gain [97].

Seven studies examined the association between sleep duration and emotional regulation in children and youth. Five studies used an RCT design. Four studies showed an effect on emotional regulation [98–101]. One study showed no effect of time in bed on mood [102]. Out of two longitudinal studies [103, 104], one study reported that longer sleep was related to better emotional regulation at follow-up [104], the other study reported that daily variability in sleep duration predicted greater symptomatology [103].

Six studies examined the association between sleep duration and cognition in children and youth. Five studies used an RCT; four of these reported that longer sleep was associated with better cognition [105–108]. One study showed no sleep duration effects on cognition [109]. One longitudinal study showed significant favourable associations between average nightly sleep duration, executive function and sedentary behaviour [110].

Three studies examined the association between sleep duration and academic achievement in children and youth. Two studies used a longitudinal design; one study reported that short sleep duration did not predict cumulative grade point averages at follow-up [111]. The other study reported nonlinear positive associations of sleep duration with grade point average and English test scores [103]. One RCT showed that extended sleep of 18.2 min per night was significantly associated with improved mathematics and English grades [112].

Three studies examined the association between sleep duration and quality of life/well-being in children and youth. These longitudinal studies reported mixed results [113–115]. Gustaffson et al. reported that longer sleep duration was associated with better overall health in 12- to 15-year-olds, but there was no association in 10-year-olds [113]. Magee et al. reported that long sleep duration was associated with a decline in physical and school functioning [114]. Price et al. reported that compared with children who had psychosocial health-related quality of life problems, children who did not slept slightly less at 6-7 years, but not 8-9 years [115].

One longitudinal study examined the association between sleep duration and cardiometabolic biomarkers in children and youth. This study reported that females who had longer sleep duration had higher levels of systolic blood pressure and diastolic blood pressure. Among males, an inverse association was found, where those who had longer sleep duration had lower levels of systolic blood pressure and diastolic blood pressure [116].

The assessed quality of overall evidence using GRADE criteria for these outcomes (“moderate” for RCTs and “very low” for longitudinal studies) did not change as a result of including these additional studies.

#### Integrated

The final systematic review update included studies that investigated combinations of physical activity, sedentary behaviour, and sleep and their association with health indicators. The updated searches yielded 168 studies, with 20 additional studies meeting the inclusion criteria for the update.

Three longitudinal studies examined the association between combinations of movement behaviours and body composition in school-aged children and youth [117–119]. According to one study, reallocation of time from sleep, sedentary behaviour or LPA to MVPA was associated with lower adiposity [118]. Another study reported that reallocation of time from sedentary behaviour to MVPA was associated with lower adiposity [120]. However, no associations were reported for reallocations from sedentary behaviour to LPA [119].

Of the cross-sectional studies, two found lower adiposity among children meeting all three guidelines (physical activity, screen time and sleep) compared to those meeting none or any one or two of these guidelines [6, 8]. One study found lower adiposity among those meeting physical activity guidelines and those meeting sleep and screen time guidelines, compared to those who were not [121]. Another study reported lower adiposity among clusters of children with high physical activity compared to clusters with combinations of low physical activity/high sleep, high screen time/low sleep or high non-screen sedentary behaviour /poor diet [122]. Three studies found children characterised by the combination of high physical activity/low sedentary behaviour had lower adiposity than those characterised by low physical activity/high sedentary behaviour [123–125]. Two 24-hour isotemporal substitution studies reported that the reallocation of time to MVPA from either sleep, sedentary behaviour or LPA was associated with lower adiposity [126, 127]. In one of these studies, the reallocation of time to LPA from sedentary behaviour was associated with lower adiposity, as was the reallocation of time to sleep from sedentary behaviour or LPA in some age groups/sexes in both the studies [126]. In the five isotemporal substitution studies [128–132] of waking activities only (not including sleep), lower adiposity was reported when time was reallocated away from sedentary behaviour and given to either: MPA, VPA or MVPA [128, 129]. Reallocations from sedentary behaviour to LPA were favourable in two studies [128, 130], but unfavourable in another study [129]. Reallocations from LPA [128, 131] or MPA [128] to VPA were associated with lower adiposity. Of two compositional data studies [133, 134], both reported lower adiposity with higher MVPA or lower LPA, each relative to remaining behaviours, while one also reported lower adiposity with higher sleep, or lower sedentary behaviour, each relative to remaining behaviours [133].

Five cross-sectional studies examined the association between combinations of movement behaviours and cardiometabolic health in children and youth [6, 135–138]. Better cardiometabolic health was reported in one study for children meeting all three guidelines (physical activity, screen time and sleep) [139] compared with children meeting none, one or two guidelines; and children meeting both physical activity and sedentary screen time guidelines, compared to those not meeting these two guidelines. One study found that, among children with high levels of SB, those with high VPA had better cholesterol markers than those with low VPA [138]. Better cardiometabolic health was reported for the reallocation of time to VPA from LPA [137], and to MVPA from sedentary behaviour or LPA [136]. No associations were seen for other reallocations. One study reported better cardiometabolic health among children with higher MVPA, relative to the remaining movement behaviours [135].

Six studies examined the associations between combinations of movement behaviours and fitness. One longitudinal study found that the reallocation of time to VPA from sedentary behaviour or LPA was associated with better fitness [140]. Of the five cross-sectional studies, one study reported better fitness among children who met all three guidelines [139]; and among children who met both physical activity and sedentary screen time guidelines, compared to those who did not meet these two guidelines. One study found children characterized by high physical activity had better fitness than groups characterized by low physical activity/ high sleep, high screen time/low sleep, or high non-screen sedentary behaviour /low sleep [141]. In another study, better fitness was associated with the reallocation of time to VPA from sedentary behaviour [140]. The remaining study reported better fitness among children with higher MVPA and with lower sedentary behaviour, relative to other movement behaviours [135].

Two cross-sectional studies examined the association between combinations of movement behaviours and health-related quality of life [142, 143]. One study reported better health-related quality of life children with higher MVPA, relative to other movement behaviours [142]. In the other study, better health-related quality of life was reported among children meeting all three guidelines (physical activity, screen time and sleep) compared with children meeting none, one or two of these guidelines; and for children meeting both the sleep and screen guidelines, compared to those not meeting these two guidelines.

Two cross-sectional studies examined the association between combinations of movement behaviours and behavioural outcomes [135, 139]. Better behavioural outcomes were reported in one study for children meeting all three guidelines (physical activity, screen time and sleep) compared with children meeting none, one or two of these guidelines; and for children meeting both the physical activity and screen time guidelines, compared to those not meeting these two guidelines [139]. In the second study, better behavioural outcomes were reported for children with higher sleep, relative to other movement behaviours [135].

The assessed quality of overall evidence using GRADE criteria for these outcomes did not change as a result of including these additional studies.

#### Consensus

The Australian Guideline Development Group reached consensus in the interpretation of the evidence for each movement behaviour and for the integration of the three behaviours. On the basis of the evidence from the systematic reviews from Canada, the Canadian GRADE tables and recommendations, and the updated systematic reviews in Australia, the Guideline Development Group adopted the Canadian recommendations.

Following the consensus that Australia would adopt the Canadian recommendations, the Guideline Development Group then discussed if the wording of the Canadian Guidelines, title and preamble was appropriate for the Australian context. As a result of this discussion, several minor changes were made to the wording of the title, preamble, and guidelines. Group members were able to suggest a change, provide a rationale for the change. This was then discussed by the group. The Guideline Development Group determined if the proposed change would be consistent with the quality and strength of the evidence recommended and ensured it would not unintentionally alter the interpretation of the guideline. Consensus was required for a change to be accepted. Table S2 summarised the changes in wording between the Canadian and Australian Guidelines. Members of the Guideline Development Group endorsed the draft title, preamble, and guidelines, that were used for the stakeholder consultations.

### Stakeholder consultations and final guidelines

The draft guidelines developed and approved by the Guideline Development Group at the August 2018 meeting were used to seek broader consultation through an online stakeholder survey, focus groups and key informant interviews. At the close of the online survey, responses from 237 participants were tabulated and analysed. The number of responses varied by question with between 186 to 237 responses for closed-ended questions. Respondents were from every state and territory in Australia with 49.5% from New South Wales, 8.1% from Victoria, 4.3% from Queensland, 7.0% from Western Australia, 7.5% from South Australia, 6.5% from the Australian Capital Territory, 0.5% from the Northern Territory, and 5.4% from Tasmania. Approximately one out of nine respondents were from outside Australia (11.3%). Respondents identified as being from the following sectors: education (49.7%), research/academia (19.8%), public health (8.0%), healthcare/services (6.4%), government (5.4%), Commonwealth/State Departments of Health (4.8%), sport (2.1%), other (2.1%), physical activity/fitness (1.1%), and recreation (0.5%).

The proportion of respondents who strongly agreed or somewhat agreed that the title, preamble, and guidelines were clearly stated was very high, ranging from 83% to 97%. The proportion who strongly agreed or somewhat agreed with the message in these sections ranged from 38% to 72%. A summary of the responses from the stakeholder survey is in Table 3. For the open-ended questions, most suggestions were related to the wording, identification of key groups for implementing the 24-Hour Movement Guidelines, and determining the support these groups would require. Forty percent of respondents were interested in supporting the Guidelines once released.

**Table 3:** Summary results of closed-ended stakeholder survey questions.

Question	Total (n)	Strongly agree, % (n)	Somewhat agree, % (n)	Combined agreement % (n)	Neither agree nor disagree, % (n)	Somewhat disagree, % (n)	Strongly disagree, % (n)
Is the title clearly stated?	237	124 (52.3)	74 (31.2)	198 (83.5)	7 (3.0)	28 (11.8)	4 (1.7)
Do you agree with the title?	235	90 (38.3)	93 (39.6)	183 (77.9)	22 (9.4)	26 (11.1)	4 (1.7)
Is the preamble clearly stated?	210	123 (58.6)	78 (37.1)	201 (95.7)	3 (1.4)	4 (1.9)	2 (1.0)
Do you agree with the preamble?	209	133 (63.6)	63 (30.1)	196 (93.8)	6 (2.9)	6 (2.9)	1 (0.5)
Would you use the preamble?	210	90 (42.9)	88 (41.9)	178 (84.8)	15 (7.1)	13 (6.2)	4 (1.9)
The 24-hour Guidelines are clearly stated	199	125 (62.8)	58 (29.2)	183 (87.1)	5 (2.5)	8 (4.0)	3 (1.5)
Do you agree with the Guidelines?	200	144 (72.0)	50 (25.0)	194 (97.0)	3 (1.5)	3 (1.5)	0 (0.0)
	Total (n)	Much more useful, % (n)	More useful, % (n)	Neutral, % (n)	Less useful, % (n)	Much less useful, % (n)	
In comparison to separate physical activity, sedentary behaviour, and sleep guidelines, do you find these integrated Guidelines…	197	65 (33.0)	97 (49.3)	162 (82.2)	31 (15.7)	4 (2.0)	
	Total (n)	Always	Frequently	Combined High Use	Occasionally	Seldom/never	
Would you use the 24-Hour Guidelines?	196	79 (39.3)	96 (47.8)	175 (87.1)	21 (10.5)	5 (2.5)	

Thirteen key informant interviews and three focus groups were conducted. The results supported the findings from the online survey. All key stakeholders unanimously agreed with the ‘integrated’ nature of the new 24-Hour Movement Guidelines. Stakeholders suggested that integrating the Guidelines made the information more accessible. Several stakeholders commented that it made sense to have them integrated as the behaviours were so closely interrelated.

All stakeholders suggested that the new 24-Hour Movement Guidelines were clearly presented and were understandable, in general, for professional and policy makers “*but not for the children themselves*” [Education Sector, ACT]. Some stakeholders suggested that they thought the prescription (i.e. the number of hours) of each behaviour was helpful.

Several stakeholders, including children and young people, suggested that the wording of the physical activity component of the Guidelines was confusing and needed to be modified. The wording of the Guideline relating to sedentary behaviour also raised some questions. Stakeholders were not clear what was meant by “long periods of time” and how this would be operationalised by children and young people. Some stakeholders suggested that additional information further highlighting the importance of sleep routines, quality of sleep as well as the relationship between the movement behaviours and broader health outcomes such as self-esteem, health and wellbeing would have been a valuable addition to the guidelines.

Irrespective of the sector, all stakeholders suggested that they would be able to use the new 24-Hour Movement Guidelines in their professional practice or in their home environment. Several suggestions to maximise their uptake were provided by the stakeholders. For example, the inclusion of “*examples of different types of physical activities*” [Education sector, NSW and VIC] or “*examples of how to limit screen time*” [Children and Young People from a number of Australian states and territories] were suggested. An explanation of some of the more complex words such as moderate- to vigorous-intensity physical activity was also suggested. For optimal use within the Education Sector, key stakeholders suggested that the Guidelines should be embedded within the Australian Curriculum and the link between the 24-Hour movement behaviours and educational outcomes and learning needed to be clear. Key stakeholders were highly conscious and aware of the already overcrowded curriculum and the high workload of staff and students. They suggested that teachers and principals were unlikely to incorporate or promote the Guidelines in their core business unless there was direct link to educational outcomes. Some stakeholders suggested that the integrated nature (i.e. having all three behaviours together) of the Guidelines could potentially result in end users feeling overwhelmed and in turn disregarding the Guidelines. Stakeholders suggested perhaps the marketing and promotional material should take on a ‘tiered approach’, inclusive of a very simple version for children and young people to a more complex version for parents and professionals.

The stakeholders suggested several dissemination options for the new 24-Hour Movement Guidelines. Most stakeholders suggested a multi-level approach that could be inclusive of flyers and brochures in community centres, gyms and health professional environment, promotion through external facilitated sport in schools, ministerial communications at both the Federal and State levels, social media campaigns, traditional media campaigns (inclusive of personal testimonies), websites, peak bodies for educators and principals, additional professional development for educators and inclusion in pre-service training.

The main dissemination avenue suggested was through parents and schools. Parents would have more influence in promoting these behaviours for children (5-12 years), while schools could have a greater impact for young people (i.e. those aged 13-17 years). As suggested previously, the direct link between the movement behaviours and children’s educational outcomes and learning would need to be the focus. Stakeholders suggested that if schools committed to promoting the Guidelines and incorporating them into all areas of learning then the evidence-base supporting the relationship between these behaviours and educational outcomes would need to be clear. If the promotional materials were optimal, a number of avenues could be used in the school environment to promote the Guidelines (e.g. newsletters, social media, health and physical education departments in schools, homeroom leaders/teachers, and school counsellors).

Given the diverse target group for the Guidelines, the importance of tailored dissemination approaches was emphasised by all stakeholders. Irrespective of the target age, stakeholders suggested that consistent messaging between families, schools and other places/people of influence was critical.

Several barriers were highlighted by stakeholders that would need to be considered in the development of promotional material. The obvious social change around smart phones and screen time has changed the nature of screen-based activities and was a consistent barrier mentioned by many stakeholders. Other barriers included the time-poor reality of parents, the over scheduled child and young person, and cost and access to facilities.

Another barrier mentioned was the media highlighting the potential risks or injuries associated with physical activity. Uncertainty around ongoing funding at State and Federal levels to support existing or new programs, such as *Ride to School* initiatives and NSW *Premier’s Be Active Challenge* was also highlighted as a barrier for further promotion of the new Guidelines.

**Fig. 2:**
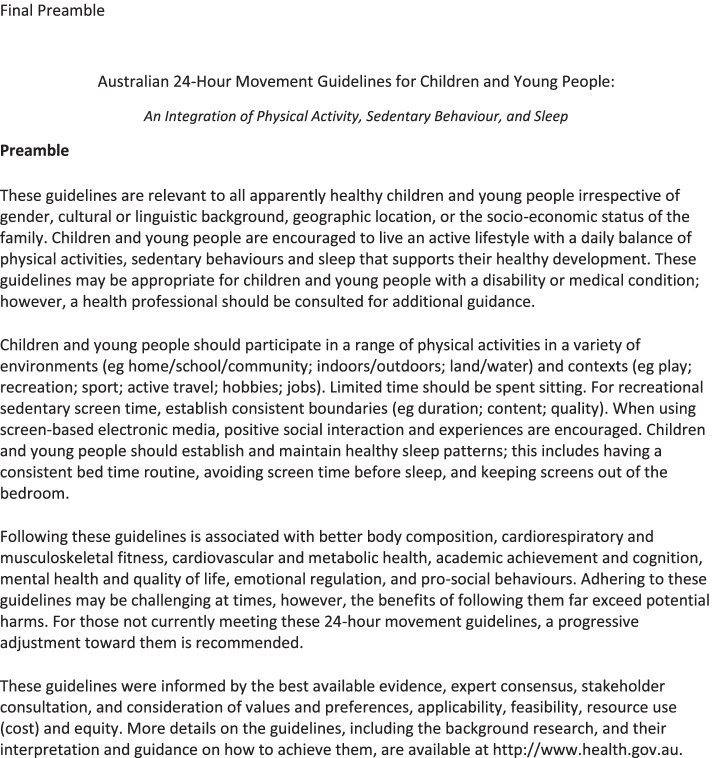
Final Preamble

**Fig. 3:**
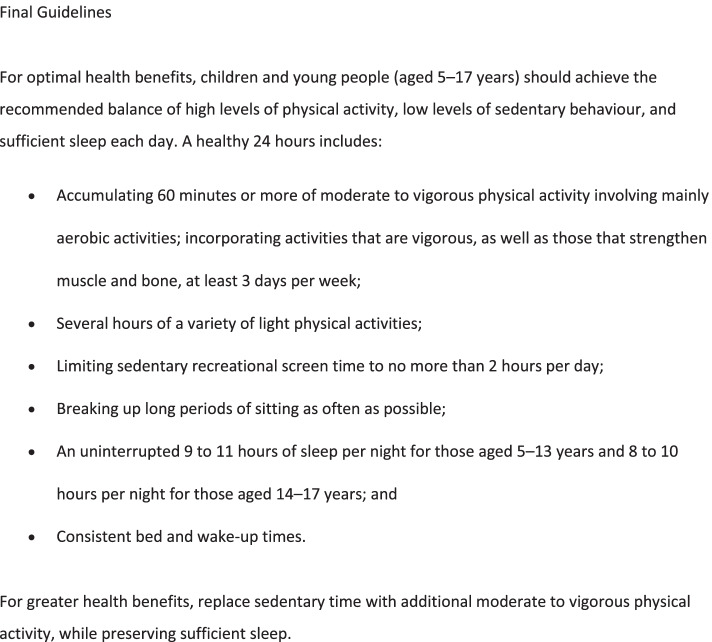
Final Guidelines

Stakeholders suggested that promoting all three movement behaviours would be an ongoing challenge. Physical activity and sedentary behaviours have been a key focus for several years, however incorporating healthy sleep behaviours into public health messaging is new. Thus, a concerted effort would be needed to ensure that all behaviours are equally promoted in the dissemination of the new Guidelines. The final guidelines, including the title and preamble, are provided in Figs. 2 and 3.

### Dissemination, implementation, and evaluation plans

A sub-group of the Guideline Development Group developed a summary of suggested dissemination and implementation activities. This included key communication strategies in the lead up to and after the official launch of the Guidelines, with government and non-government support for the integration of the guidelines into schools, primary and allied health services, sport and recreation, and as part of whole-of-government approaches. Consultation with key users of the Guidelines during the Guideline Development Group meeting indicated that targeting parents and schools using a multi-level approach would be most beneficial in enhancing awareness and adoption of the guidelines. A “world-café” workshop [144, 145] was conducted as part of the Guideline Development Group meeting to brainstorm communication, dissemination, and implementation activities in relation to media and complementary packages, training, and scaling-up of proven programs.

### Research gaps and surveillance recommendations

Research gaps were identified through the updates of the systematic reviews and during discussions at the Guideline Development Group meeting. This included thinking about surveillance and monitoring of the new guidelines. The full set of research gaps were distributed to the Guideline Development Group after the meeting for further feedback and agreement (summarised in Table S3).

A surveillance sub-committee was established at the Guideline Development Group meeting and tasked with recommending questions/methods that could be used for surveillance and monitoring of the Guidelines. This sub-committee met three times via teleconference. This committee included representatives from Sport Australia and the Australian Bureau of Statistics, the two organisations who routinely collect nationally representative data on physical activity among children and young people. The sub-committee recommended physical activity, sedentary behaviour, and sleep questions that could be incorporated into the AusPlay Survey (Sport Australia), an interview-administered telephone questionnaire for 5- to 14-year-old children and a self-report for 15- to 17-year-olds. These questions are shown in Table S4.

## Discussion

This paper describes the process to develop the *Australian 24-Hour Movement Guidelines for Children and Young People (5 to 17 years): An Integration of Physical Activity, Sedentary Behaviour, and Sleep* and the outcome. The evolution from separate guidelines for each of these behaviours to integrated guidelines for this age group is relatively new. Feedback on the integrated approach for this age group was well received by key stakeholders. The Australian Guideline Development Group was positive in their response to the task of developing integrated guidelines. This was aided by having the *Canadian 24-Hour Movement Guidelines for Children and Youth* to refer to and the presence of panel members who were experienced with the 24-Hour approach to guideline development. The Australian guideline development followed the GRADE-ADOLOPMENT process. A strength of the guideline development process was the multidisciplinary composition of the Guideline Development Group, which included content experts in all the movement behaviours, clinicians, policy making, evidence synthesis and health economics experts, and key stakeholders from the education, sport, disability, transportation, and multicultural sectors. Involvement of international experts (Canada, USA, South Africa) provided an opportunity to learn from other countries and consider harmonisation of guidelines across countries.

The GRADE-ADOLOPMENT approach [146] was used to update the Australian Guidelines. Following this process, the *Canadian 24-Hour Movement Guidelines for Children and Youth* were adopted. This allowed the Australian guidelines to be updated over a shorter period and much less of a cost than if the full GRADE approach was followed. We recommend the GRADE-ADOLOPMENT approach if a credible set of guidelines and related materials (e.g., PICOs) are available. This approach has been used to develop national movement behaviour guidelines in several countries.

The Leadership Group slightly modified the sedentary behaviour PICO from that used in Canada. Psychological distress was added as a critical outcome and self-esteem moved from an important to critical outcome. This resulted in the development of a new systematic review for psychological distress, with no date limit. While somewhat challenging given the timeline for the project, it was achievable. The Leadership Group also discussed whether to include cross-sectional studies in the updated systematic reviews. It was decided that even if several studies were found, the level of evidence would unlikely be enough to change the overall recommendation. As it was highly likely studies using these designs would not make a difference the Leadership Group decided not to change this in the PICO, with the exception of the systematic review on combinations of movement behaviours, where because of the small number of studies likely to be found, it was decided to include cross-sectional studies. This decision was somewhat influenced by the limited timeframe and resources available. In future, it would be optimal if there was an initial face-to-face meeting of the Guideline Development Group to discuss existing PICOs from other guidelines and their appropriateness for the Australian context rather than the Leadership Group completing this task via email and teleconference. Due to the size of the Guideline Development Group, the project budget, and the timeline, which were parameters of the project set by the funding source, it was not feasible for a face-to-face meeting to be held for this task. Conducting the meeting online is also a possibility.

While these new guidelines integrated physical activity, sedentary behaviour and sleep into a 24-hour approach, the guidelines for physical activity and sedentary behaviour were identical to the previous *Australian Physical Activity Guidelines for Children and Young People* *[*3*]* *and the Australian Sedentary Behaviour Guidelines for Children and Young People* *[*2*]*. In the preamble, the main change was the inclusion of a statement regarding the importance of healthy sleep patterns and means of encouraging sleep. A contextual statement was added establishing the importance of consistent boundaries for recreational sedentary screen time and to encourage positive social interaction and quality experiences when using screen-based electronic media. The term “geographic location” was added to the list of sociodemographic descriptors to indicate that the guidelines apply to children in urban, regional, and remote areas of Australia. Finally, there is a statement summarising what sources of data were used to inform the guidelines and what considerations were made. Compared with former guidelines, the main change was the integration of all movement behaviours across a 24-hour period, with a specific statement at the end of the guidelines outlining how to do this for greater health benefits. We replaced the term “electronic media for entertainment” with “sedentary recreational screen time” to clarify that the evidence upon which this guideline is based comes largely from studies where participants were likely to be sedentary when engaged with screens. It is important to reiterate the focus on recreational not educational screen time and to acknowledge that educational use is determined by pedagogical expertise for quite distinctive purposes. The amount of light-intensity physical activity has also been somewhat quantified (*several* hours, which could be operationalised as at least three hours) based on new evidence that allowed us to somewhat quantify a duration for this behaviour.

Although presented as “24-Hour Movement Guidelines”, they are not prescriptive recommendations that summate to 24 hours, (e.g., for children aged 5-12 years, at least 1 hour of MVPA and up to several hours of light-intensity physical activity, no more than 2 hours of sedentary recreational screen time, and 9-11 hours of sleep). For example, if one child sleeps 11 hours and another 9 hours, the latter has two additional hours of time to be distributed among the wake-time behaviours. In addition, some degree of day-to-day variability, such as that across a week given different activities on different days, is normal and provision of ranges allows for this flexibility. For these reasons, and to be accommodating to different schedules and changes in schedules, the guidelines provide broader recommendations, such as “replace sedentary time with additional moderate- to vigorous-intensity physical activity” and “breaking up long periods of sitting as often as possible” to give directional advice while recognising the dynamics of the component behaviours between and within individuals. Collectively, guidance for healthy movement behaviours over the whole day is provided.

### Release of the guidelines, dissemination, implementation, integration, and evaluation planning

The Guidelines were officially released by the Federal Minister for Sport in Canberra, Australian Capital Territory, on the 4^th^ April 2019. Accompanying the launch were brochures and posters targeting parents and educators (teachers, coaches, mentors, instructors) and social media posts from the Minister’s office using the hashtags #SleepMovePlay and #movementguidelines. An online version of the brochure can be found on the Australian Government Department of Health website. In the lead up to the launch, members of the Guideline Development Group were approached to be spokespeople for the guidelines for their state/territory. This sub-group met several times via teleconference with the Department of Health to plan for the launch, comment on the promotional materials and social media strategy, and finalise the media release. In the three-week period following the launch (April 3 to April 23 2019) there were 17 online editorial mentions and 65 broadcast editorial mentions of the guidelines, with a potential reach of 8 million readers/viewers. The net tonality score was +100, indicating 100% positive mentions of the guidelines in the media. Social media analysis revealed the launch earned 13,300 impressions on Twitter. In late June 2019, the Australian Department of Health funded additional Facebook paid posts targeting parents and educators with children aged 5 to 17 years. These were supplemented with Tweets from the Department of Health which earned over 4,000 impressions. A plan was developed for a comprehensive evaluation of the subsequent dissemination, implementation, and integration activities to assess community ownership and population-level community impacts on children’s and young people’s movement behaviours over time.

### Updating the guidelines

The final stage in the guideline development process is the planning of updates and revisions [26]. The Australian Guideline Development Group recommends that these guidelines be reviewed, and updated if necessary, at least every 10 years or when significant new research emerges warranting change.

## Conclusion

This is the second time, to our knowledge, the GRADE-ADOLOPMENT approach has been used to develop national guidelines for children and young people. Based on this approach, the Australian Guideline Development Group’s judgements did not warrant changing the strength and directions of the recommendations. Consequently, the Canadian recommendations were adopted. The *Australian 24-Hour Movement Guidelines for Children and Young People: An Integration of Physical Activity, Sedentary Behaviour, and Sleep* represents a paradigm shift in how movement behaviours among our children and youth are operationalised. The evidence we reviewed supports that childhood and adolescent health can be enhanced through the recommended amounts of physical activity, sedentary behaviour, and sleep each day. Meeting the recommendations –individually and in combination – is associated with better health in children and young people. These benefits far outweigh any potential harms. These guidelines are relevant for apparently healthy children and young people. It is hoped that participating in healthy physical activity, sedentary, and sleep behaviours during childhood and adolescence will result in immediate and long-term health benefits and establish lifestyle habits that can be sustained into adulthood.

## Supplementary Information

**Additional file 1.**

## Data Availability

Any raw data or materials used in the preparation of this manuscript are available upon reasonable request to Professor Anthony Okely (tokely@uow.edu.au).

## Abbreviations

ADOLOPMENT: A methodology that combines the advantages of adoption, adaptation, and de novo development of recommendations based on the GRADE EtD frameworks
AGREE: Appraisal of Guidelines for Research & Evaluation
ARC: Australian Research Council
EtD: Evidence-to-decision frameworks
GRADE: Grading of Recommendations Assessment, Development, and Evaluation
LPA: Light-intensity physical activity
MVPA: Moderate- to Vigorous-intensity Physical Activity
NHMRC: National Health & Medical Research Council
NSW: New South Wales
PA: Physical activity
PICO: Population, Intervention, Comparator, and Outcome
PROSPRO: The Prospective Register of Systematic Reviews
SB: Sedentary behavior
UOW: University of Wollongong
