# Supplementary material for: A collaborative approach to adopting/adapting guidelines. The Australian 24-hour movement guidelines for children (5-12 years) and young people (13-17 years): An integration of physical activity, sedentary behaviour, and sleep
Source: Int J Behav Nutr Phys Act. 2022 Jan 6;19:2. doi: 10.1186/s12966-021-01236-2 (PMC8734238; doi:10.1186/s12966-021-01236-2)
Supplement: Supplementary file 1 — Additional file 1. [file 12966_2021_1236_MOESM1_ESM.docx]

**Supplementary file**

Table S1. Guideline Development Group

| Panel Member | Affiliation | Role | Conflict of Interest Declaration |
| --- | --- | --- | --- |
| **Research Experts** |  |  |  |
| Anthony Okely | University of Wollongong, Wollongong, Australia | Chair, researcher, content expert PA, SB | - Member of Consensus Committee for Canadian 24-hour integrated movement guidelines for the early years  - Member of Consensus Committee for Canadian 24-hour integrated movement guidelines for children and youth  - Member of Guideline Development Group, World Health Organization Physical Activity, Sedentary and Sleep Behaviour Guidelines for Children Birth to 5.  - Member of Guideline Development Group, UK Chief Medical Officer’s Physical Activity, Sedentary and Sleep Behaviour Guidelines for Children Birth to 5  - Member of Guideline Development Group, South African Physical Activity, Sedentary and Sleep Behaviour Guidelines for Children Birth to 5  - Paid consultancy from Foxtel on active interstitials for children’s pay television channels.  - Consultancy to Early Childhood Australia to deliver Munch & Move Professional Development for early childhood educators in NSW.  *- Paid consultancy from Foxtel to advise on inclusion of physical activity interstitials (activity breaks during advertising periods) on children’s pay television channels*  *- My expenses were covered to attend meetings for Canadian 24-Hour movement guidelines for children and youth in 2016 and 2017 (2 times).* |
| Sarah Loughran | University of Wollongong, Wollongong, Australia | researcher, expert Sleep | - Member of the leadership group for the Australian 24-Hour Movement Guidelines for the Early Years  - Has received funding from the NHMRC for research studies focused on sleep, screen time, and mobile phone use in children  - Has published journal articles and given presentations on sleep, screen time, and mobile phone use in children |
| Dylan Cliff | University of Wollongong, Wollongong, Australia | researcher, expert SB and PA | - Leadership group member for the Australian 24-Hour Movement Guidelines for the Early Years.  - Has received funding from the ARC, NHMRC and National Heart Foundation for research studies focused on physical activity, sedentary behaviour, and electronic media use in children.  - Has published journal articles and given presentations on physical activity, sedentary behaviour, electronic media use and sleep in children.  - Has provided consultancy to Early Childhood Australia and NSW Health to deliver Munch & Move Professional Development for educators in NSW related to physical activity promotion for children. |
| Tim Olds | University of South Australia, Adelaide, Australia | expert sleep, compositional analyses | - Grant: NHMRC Project Grant APP143379 (2018-2021). *Life On Holidays: Fitness lost, fatness regained?*  - Grant: A Better Start National Science Challenge and Cure Kids (2017-2019). Childhood Obesity: Data to Action (CODA): Translation ehaviour to inform an evidence-based childhood intervention obesity agenda.  - Grant: ARC Discovery Indigenous IN160100035 (2016-2018). *Participation in sport and its links to education attainment and health and wellbeing.*  - Grant: NHMRC Project Grant APP1109355 (2016-2019). *Biological, phenotypic and public health costs of risk and protective pathways to* *non-communicable disease in children and adults: The national Longitudinal Study of Australian Children*  - Grant: NHMRC Project Grant APP1080186 (2015-2018). *Active Team – Examining an online social networking intervention to increase physical activity in controlled (RCT) and ecological (ET) settings* |
| Anne-Maree Parrish | University of Wollongong, Wollongong, Australia | Researcher, expert PA, SB | - Has received funding from the National Heart Foundation for research focusing on physical activity and sedentary behaviour in children and youth  - Has published journals and given conference presentations on physical activity and sedentary behaviour in children and youth  - Has been involved in an evaluation for the NSW Department of Education which assessed sedentary time in children and youth  Is a committee member of the Public Health Association of Australia’s Health Promotion Special Interest group have revised the physical activity policy as part of this role.  - Has been involved in the development of the existing Australian Physical Activity and Sedentary Behaviour Guidelines for Australian Children and youth |
| Rachel Jones | University of Wollongong, Wollongong, Australia | researcher, expert knowledge translation | - Member of Consensus Committee for Australian 24-hour integrated movement guidelines for the early years (Stakeholder Consultations and dissemination)  - Consultancy to Early Childhood Australia to deliver Munch & Move Professional Development for early childhood educators in NSW.  - Has received funding from the NHMRC and National Heart Foundation for research studies focused on physical activity and sedentary behaviour in children.  - Has published journal articles and given presentations on physical activity and sedentary behaviour in children.  - Co-lead – Special Interest Group Early Childhood and Care, ISBNPA |
| Rebecca Stanley | University of Wollongong, Wollongong, Australia | researcher, expert stakeholder consultation | - Member of Consensus Committee for Australian 24-hour integrated movement guidelines for the early years (leadership of the Stakeholder Consultations)  - Consultancy to Early Childhood Australia to deliver Munch & Move Professional Development for early childhood educators in NSW.  - Funded by a NSW Health Early-Mid Career Research Fellowship to conduct research on physical activity and sedentary behaviours of Australian Indigenous children.  - Grant – Early Start Research Institute – Okely, A, Rissel, C, Williams, M, Franco, L, Furber S, **Stanley RM**, Kelly, B, Feng X, F, Roberto Dos Santos, R, Cliff, D, Jones, R, Hammersley, M, Probst, Y & Morris, J, (2017-2020) “Obesity prevention”, NSW Health Prevention Research Support Program Round 5.  - Grant – **Stanley RM**, McKnight, A, Crowe, R, Probst, Phillipson, L, Okely AD. (2015-2018), “Stronger culture, healthier lifestyles: the development of an afterschool cultural and activity program for Aboriginal children living in the Shoalhaven”, UOW Global Challenges Project Grant  - Co-chair of the International Society of Behavioural Nutrition and Physical Activity Children and Families Special Interest Group. |
| Lisa Kervin | University of Wollongong, Wollongong, Australia | digital literacy | Has funded research to investigate young children’s use of digital technology:   - Verenikina, I., Kervin, L. & Murphy, C. Conceptualising digital play: The role of tablet technologies in the development of imaginative play of young children. ARC Discovery DP140100328 - Kervin, L. & Mantei, L. Examining digital reading practices of emerging readers URC Partnership Grant (with Catholic Education, Diocese of Wollongong) ($10, 000 URC funding, $22, 000 cash and in-kind CEO) 2012-2013   - Has published journal articles and chapters reporting on young children’s use of digital technology.  - Is involved (as one of 6 Cis) on a current ARC Centre of Excellence bid. |
| Zhiguang Zhang | University of Wollongong, Wollongong, Australia | PhD student for systematic review of sedentary behaviour | Funded through an PhD scholarship from the China Scholarship Council and an International Postgraduate Tuition Award from the University of Wollongong. |
| Sandra Downie | Preventive Programs, Commonwealth Department of Health | stakeholder, end user, government | Nothing to declare |
| Adam Verrender | University of Wollongong, Wollongong, Australia | PhD student for systematic review on sleep | - Member of the Guideline Development Group for the Australian 24-hour movement guidelines for children of the Early Years. |
| **Stakeholder Group and Knowledge Users** |  |  |  |
| Jo Salmon | Deakin University | content expert physical activity and sedentary behaviour | - My spouse manufactures height adjustable desks for schools. |
| Trevor Shilton | National Heart Foundation of Australia, Perth, Australia | stakeholder, cardiovascular health, messaging | - Member of the Board. International Society for Physical Activity and Health (ISPAH)  - Chairman of Global Advocacy for Physical Activity (GAPA), the advocacy Council of ISPAH  - Member of the Board and Global Vice President for Partnerships, International Union for Health Promotion and Education (IUHPE)  - Adjunct Professor, School of Public Health, Curtin University, Bentley Western Australia  - Adjunct Associate Professor, School of Exercise Science and Health, University of Western Australia, Crawley, WA  - Member of the World Health Organization, Civil Society Working Group for the United National HighLevel Meeting on Non-communicable Disease. |
| Clair Bannerman | Department of Education, Canberra, Australia | stakeholder | Nothing to declare |
| Tamie Needham | Department of Health, NT, Australia | stakeholder | Nothing to declare |
| Elaine Marshall | Department of Health, TAS, Australia | stakeholder | - Contribute written content to state government reports, ministerial advice and submissions that contain information about children’s physical activity and sedentary behaviour.  - Working group member of a project funded through NHMRC partnership project grants scheme. |
| Jordy Kaufman | Swinburne University, VIC, Australia | content expert screen time | - Has published journal articles on children’s use of screen-based media  - He was the lead investigator on a project evaluating the ELLA apps created by the Department of Education and Training.  - Has led a literature review for the Department of Education and Training on the implications of technology use by children. The focus was on educational outcomes in the school setting, but it did touch on home use and health outcomes.  - Gave feedback on a draft of the ECA statement being developed. |
| Layne Brown | University of Wollongong, Wollongong, Australia | content expert Indigenous health | Nothing to declare |
| Janecke Wille | Federation of Ethnic Communities’ Council of Australia (FECCA) | stakeholder | Nothing to declare |
| Greg Wood | Australia Sports Commision | stakeholder | Nothing to declare |
| David Lubans | University of Newcastle, Newcastle, Australia | content expert physical activity and sedentary behaviour | Nothing to declare |
| Stuart Biddle | University of Southern Queensland, Australia | content expert physical activity, sedentary behaviour, and mental health | - Funding was received in 2016 for consultancy work for Halpern PR Limited (for an international deodorant company) concerning light intensity physical activity, primarily in adults.  - Member, International Advisory Panel, Get Britain Standing.  - Member, Expert Working Group for ‘Sedentary Behaviour’, 2017-2018 update of the UK CMO physical activity guidelines.  - Member, Expert Working Group for ‘Physical Activity and Disability’, 2017-2018update of the UK CMO physical activity guidelines. |
| Shane Pill | The Australian Council for Health, Physical Education and Recreation (ACHPER) National, SA | Representative of the health and physical education profession | Nothing to declare |
| Anthea Hargreaves | Cycling and Walking Australia New Zealand | active transportation | - Lobbying activities: In my role as Bicycle Network’s General Manager, Public Affairs I am responsible for advocating for funding and bike infrastructure at all levels of government.  - I am also the national spokesperson for Bicycle Network’s Ride2School program.  - I don’t believe there are any other activities which may impact my impartiality. |
| Nathalie Jonas | Australian Curriculum Assessment and Reporting Authority (ACARA) | Curriculum expert, health and physical education | Vice President (volunteer role): Early Childhood Organisation of South Australia. |
| Natasha Schranz | Active Healthy Kids Australia, University of South Australia and National Heart Foundation (SA) | physical activity and dissemination of guidelines | Nothing to declare |
| Perry Campbell | Australian Children’s Education and Care Quality Authority (ACECQA) | education, quality assurance | Nothing to declare |
| Karen Ingram/Hayley Dean* | NSW Education Standards Authority (NESA) | curriculum experts, health and physical education | Nothing to declare |
| Kar Hau Chong | University of Wollongong | PhD student for systematic review on physical activity | Recipient of a University Postgraduate Award and an International Postgraduate Tuition Award at the University of Wollongong |
| Dorothea Dumuid | University of South Australia | post-doctoral fellow, integrated review of movement behaviours | - Supported by NHMRC (APP1162166) /Heart Foundation (102084) Fellowship (2019-2022)  - Australian Government Research Training Program Scholarship (2015-2018)  - NHMRC Grants (APP1171313; APP1163338)  - Director and physiotherapist at Have a Ball Children’s Physiotherapy Services (2015-2018) |
| Hayley Lewthwaite | University of South Australia | post-doctoral fellow, integrated review of behaviours | Travel scholarship, University of South Australia  Australian Government Research Training Program Scholarship |
| Natalie Toms | Preventive Programs, Commonwealth Department of Health, Canberra, Australia | stakeholder, end user | Nothing to declare |
| **International Collaborators** |  |  |  |
| Mark Tremblay | Children’s Hospital of Eastern Ontario Research Institute, Ottawa, Canada | chair of Canadian Guideline Panel, researcher, content expert physical activity, sedentary behaviour | - I was involved in the development of the existing Australian Physical Activity and Sedentary Behaviour Guidelines for Australian Children and Youth  - I led the development of the Canadian 24-Hour Movement Guidelines for Children and Youth, and it is possible these guidelines will be involved in the Australian adolopment process.  - I have been involved in the development of many other physical activity, sedentary behaviour and 24-hour movement guidelines for additional age groups (e.g., early years, adults, older adults, pregnancy) in Canada and in other countries  - I was Chair of the Canadian Society for Exercise Physiology Canadian Guideline Committee for many years  - I have held several grants for the development of the Canadian Guidelines from the Canadian Institutes of Health Research, the Public Health Agency of Canada, the Conference Board of Canada, ParticipACTION, the University of Alberta, and the Canadian Society for Exercise Physiology — no research monies were used to pay me directly |
| Peter Katzmarzyk | Pennington Biomedical Research Center, USA | International expert, Member of US PA Guidelines Advisory Group | Nothing to declare |
| Catherine Draper | South African Medical Research Council Developmental Pathways for Health Research Unit University of the Witwatersrand, Johannesburg, South Africa | International expert, Chair of South African Guideline Panel | Nothing to declare |
| **Methodology Consultants and Project Management** |  |  |  |
| Davina Ghersi | NHMRC (Canberra), Australia | GRADE-ADOLPMENT, AGREE methodology expert | - Employee of National Health and Medical Research Council as Senior Principal Research Scientists  - I also provide advice to WHO, in relation to Guidelines, specifically in nutrition.  - Member of GRADE working group |
| Simon Eckermann | University of Wollongong, Wollongong, Australia | health economist | - Member of Consensus Committee for Australian 24-hour integrated movement guidelines for the early years  - Member (health economics expert adviser) for Food Standards Australia New Zealand (FSANZ) Social Science Economics Advisory Group (SSEAG). |
| Julie Sherring | University of Wollongong, Wollongong, Australia | project management | Nothing to declare |
| Yvonne Ellis | University of Wollongong, Wollongong, Australia | project management | Nothing to declare |

*role was shared between these two panel members

**List of Acronyms**

ACECQA: Australian Children’s Education & Care Quality Authority

ARC: Australian Research Council

ECA: Early Childhood Australia

NHMRC: National Health & Medical Research Council

NSW: New South Wales

UOW: University of Wollongong

**Table S2. Differences in the Australian Guidelines compared with the Canadian Guidelines**

| **Canadian Guidelines (Original)** | **Australian Guidelines** | **Reasoning** |
| --- | --- | --- |
| **Title** | | |
| ***Canadian*** 24-Hour Movement Guidelines for Children and **Youth**: *An Integration of Physical Activity, Sedentary Behaviour and Sleep.* | ***Australian*** 24-Hour Movement Guidelines for Children and **Young People** (5 – 17 years): *An Integration of Physical Activity, Sedentary Behaviour, and Sleep.* | To identify the relevant country and age group.  Young people was used to keep the term consistent with the previous to maintain consistency in wording with the previous Australian Physical Activity and Sedentary Behaviour Guidelines for Children and Young People |
| **Preamble** | | |
| These guidelines are relevant to all apparently healthy children and youth (aged 5-17 years***)***, irrespective of gender, race, ethnicity, or the socio-economic status of the family. | These guidelines are relevant to all apparently healthy children and young people irrespective of gender, cultural or **linguistic** background, **geographic location**, or the socio-economic status of the family. | This wording was more suited to the Australian context and, in the case of geographic location, consistent with wording in the Preamble of the Australian 24-Hour Movement Guidelines for the Early Years. |
| Children and youth should practice healthy sleep hygiene (habits and practices that are conducive to sleeping well), limit sedentary behaviours (especially screen time), and participate in a range of physical activities in a variety of environments (e.g., home/school/community; indoors/outdoors; land/water; **summer/winter**) and contexts (e.g., play, recreation, sport, active **transportation**, hobbies, and **chores**). For those not currently meeting these 24-hour movement guidelines, a progressive adjustment toward them is recommended.  Following these guidelines is associated with better body composition, cardiorespiratory and musculoskeletal fitness, academic achievement and cognition, emotional regulation, pro-social behaviours, cardiovascular and metabolic health, and overall quality of life. The benefits of following these guidelines far exceed potential risks.  These guidelines may be appropriate for children and youth with a disability or medical condition: however, a health professional should be consulted for additional guidance.  The specific guidelines and more details on the background research informing them, their interpretation, guidance on how to achieve them, and recommendations for research and surveillance are available at www.csep.ca/guidelines | Children and young people should participate in a range of physical activities in a variety of environments (eg home/school/community; indoors/outdoors; land/water) and contexts (eg play; recreation; sport; active **travel**; hobbies; **jobs**). Limited time should be spent sitting. **For recreational sedentary screen time, establish consistent boundaries (eg duration; content; quality). When using screen-based electronic media, positive social interaction and experiences are encouraged.** Children and young people should establish and maintain healthy sleep patterns; **this includes having a consistent bed time routine, avoiding screen time before sleep, and keeping screens out of the bedroom**.  Following these guidelines is associated with better body composition, cardiorespiratory and musculoskeletal fitness, cardiovascular and metabolic health, academic achievement and cognition, **mental health** and quality of life, emotional regulation, and pro-social behaviours. **Adhering to these guidelines may be challenging at times**, however, the benefits of following them far exceed potential harms. For those not currently meeting these 24-hour movement guidelines, a progressive adjustment toward them is recommended.  These guidelines may be appropriate for children and young people with a disability or medical condition; however, a health professional should be consulted for additional guidance.  These guidelines were informed by the best available evidence, expert consensus, stakeholder consultation, and consideration of values and preferences, applicability, feasibility, resource use (cost) and equity. More details on the guidelines, including the background research, and their interpretation and guidance on how to achieve them, are available at <http://www.health.gov.au>. | Minor wording changes, such as active travel instead of transportation and jobs instead of chores reflect variations in preferred terminology between countries.  The Guideline Development Group felt it was important to provide guidance around positive engagement in recreational sedentary screen time and in the context of ensuring healthy sleep patterns.  Avoiding screen time before sleep and keeping screens out of bedrooms is consistent with wording in the Preamble of the Australian 24-Hour Movement Guidelines for the Early Years.  Mental health was included as a result of this indicator being included in the Australian review.  The addition of the words Adhering to these guidelines may be challenging at times was included to be consistent with wording in the Preamble of the Australian 24-Hour Movement Guidelines for the Early Years |
| **Guidelines** | | |
| For optimal health benefits, children and youth (aged 5–17 years) should achieve high levels of physical activity, low levels of sedentary behaviour, and sufficient sleep each day.  A healthy 24 hours includes:   - An accumulation of at least 60 minutes per day of moderate to vigorous physical activity involving a variety of aerobic activities. Vigorous physical activities and muscle and bone strengthening activities should each be incorporated at least 3 days per week; - Several hours of a variety of structured **and unstructured** light physical activities - Uninterrupted 9 to 11 hours of sleep per night for those aged 5–13 years and 8 to 10 hours per night for those aged 14–17 years, with consistent bed and wake-up times; - No more than 2 hours per day of recreational screen time; - **Limited sitting for extended periods.** | For optimal health benefits, children and young people (aged 5–17 years) should achieve the recommended balance of high levels of physical activity, low levels of sedentary behaviour, and sufficient sleep each day.  A healthy 24 hours includes:   - Accumulating 60 minutes or more of moderate to vigorous physical activity per day involving mainly aerobic activities; incorporating activities that are vigorous, as well as those that strengthen muscle and bone, at least 3 days per week; - Several hours of a variety of light physical activities; - Limiting sedentary recreational screen time to no more than 2 hours per day; - **Breaking up long periods of sitting as often as possible;** - An uninterrupted 9 to 11 hours of sleep per night for those aged 5–13 years and 8 to 10 hours per night for those aged 14–17 years; and - Consistent bed and wake-up times. | The Australian Guideline Development Group preferred the statement Breaking up long periods of sitting as often as possible to maintain consistency in wording with the previous Australian Sedentary Behaviour Guidelines for Children and Young People |
| Preserving sufficient sleep, trading indoor time for outdoor time, and replacing sedentary behaviours and light physical activity with additional moderate to vigorous physical activity can provide greater health benefits. | For greater health benefits, replace sedentary time with additional moderate to vigorous physical activity, while preserving sufficient sleep |  |

**Table S3. Summary of research needs to address gaps in relation to the development of 24-hour integrated movement guidelines for children and young people**

| Research needs |
| --- |
| **There is a need for more high-quality studies with a particular focus on studies that:**   - examine the entire 24-hour day and physical activity, sedentary behaviour and sleep duration in children and young people; - establish standardised procedures and objective measurement to enable comparison between studies; - study a broader range of health indicators, including additional indicators of motor, cognitive and psychosocial development and the long-term effects of early interventions; - examine contemporary screen time (e.g., social media, etc) and types of screen time (Entertainment vs Communication vs Education); - use direct objective measures of sitting (i.e., thigh-mounted activity monitors) and sleeping; - examine the use of screen time measures with established psychometrics; - provide a cost-effectiveness analysis of interventions to improve physical activity, sedentary behaviours and sleep duration in children and young people; - examine the impact of sedentary screen-based activities compared with interactive sedentary screen-based activities on health indicators; - examine the relationship between sleep duration and motor development, growth and harms or injuries; - consider confounders such as diet; - use narrower age groups that align with the current sleep duration recommendations; - examine sleep quality, sleep efficiency, sleep timing (bed/wake times, napping), sleep architecture, sleep consistency, and sleep consolidation using longitudinal and intervention studies (24-Hour); - examine the best mix of activities for individual health outcomes; - examine the best mix of activities for overall health and well-being in “at-risk” populations (overweight/obese); and - examine the key factors that enable dissemination, adaptation, activation, implementation and uptake of the guidelines. |

**Table S4. Draft guideline surveillance questions for children and young people**

| **Physical Activity** | |
| --- | --- |
| 5-14 years | Physical activity is any activity that increases [(child's name)’s] heart rate and makes [him/her] get out of breath some of the time. Physical activity can be done in sports, school activities, playing with friends, or walking to school.  Interviewer: Some more examples of physical activity include brisk walking, riding, skateboarding or scooting safely to school or other places…  ...or washing the car, walking the dog, or helping to dig in the garden  *For the next question, add up all the time [child’s name] spent in physical activity each day.*  *Over the past 7 days, on how many days was [child’s name] physically active for a total of at least 60 minutes per day?*  *Some examples of activities which strengthen muscles and bones are skipping, running, hopping and jumping; climbing or swinging on monkey bars or climbing frames; playing games like tug-o-war and hopscotch or doing structured activities like dance, gymnastics and martial arts.*  *During the past 7 days, on how many days did [child’s name] do activities to strengthen [his/her] muscles and bones?* |
| 15-17 years | Physical activity is any activity that increases your heart rate and makes you get out of breath some of the time. Physical activity can be done in sports, school activities, playing with friends, or walking to school.  Interviewer: Some more examples of physical activity include recreational swimming, social tennis, fast walking, dancing or bike riding; jogging, aerobics, fast cycling, circuit training, or organised sports like basketball, soccer or netball.  *For the next question, add up all the time you spent in physical activity each day.*  *Over the past 7 days, on how many days were you physically active for a total of at least 60 minutes per day?*  *Interviewer: Some examples of activities which strengthen your muscles and bones include push ups, sit-ups, lifting weights, lunges and squats; dance; martial arts or aerobics class.*  *During the past 7 days, on how many days did you do activities to strengthen your muscles and bones?* |
| **Sedentary Behaviour** | |
| 5-14 years | Sitting or lying down, (with the exception of sleeping), are what we call ‘sedentary’ behaviours. You can be sedentary at work, at school, at home, when travelling or during leisure time. Sedentary behaviour requires little energy expenditure. Examples of sedentary behaviour include:   - Sitting or lying down while watching television or playing electronic games. - Sitting while being a passenger in a vehicle, or while travelling on a bus or train. - Sitting or lying down to read, study, write, or work at a desk or computer.   *For the next question, please add up all the time [child’s name] spent watching TV/ videos/Internet using a smart phone or tablet or playing video or computer games for entertainment each day*  *Over the past 7 days,* *on how many days did [child’s name] watch TV/ videos/ Internet using a smart phone or tablet or play video or computer games for entertainment for less than two hours while sitting or lying down?* |
| 15-17 years | Sitting or lying down, (with the exception of sleeping), are what we call ‘sedentary’ behaviours. You can be sedentary at work, at school, at home, when travelling or during leisure time. Sedentary behaviour requires little energy expenditure. Examples of sedentary behaviour include:   - Sitting or lying down while watching television or playing electronic games. - Sitting while being a passenger in a vehicle, or while travelling on a bus or train. - Sitting or lying down to read, study, write, or work at a desk or computer.   *For the next question, please add up all the time you spent watching TV/ videos/Internet using a smart phone or tablet or playing video or computer games for entertainment each day.*  *Over the past 7 days,* *on how many days did you watch TV/ videos/ Internet using a smart phone or tablet or play video or computer games for entertainment for less than two hours while sitting or lying down?* |
| **Sleep** | |
| 5-14 years | *What time did [child’s name] go to bed and turn the lights out to go to sleep last night?*  *What time did [child’s name] wake up today?* |
| 15-17 years | *What time did you go to bed and turn the lights out to go to sleep last night?*  *What time did you wake up today?* |
